# Indoor Positioning Algorithm Based on the Improved RSSI Distance Model

**DOI:** 10.3390/s18092820

**Published:** 2018-08-27

**Authors:** Guoquan Li, Enxu Geng, Zhouyang Ye, Yongjun Xu, Jinzhao Lin, Yu Pang

**Affiliations:** 1 School of Communication and Information Engineering, Chongqing University of Posts and Telecommunications, Chongqing 400065, China; ligq@cqupt.edu.cn (G.L.); S160131039@stu.cqupt.edu.cn (E.G.); 2 Chongqing Key Laboratory of Photoelectronic Information Sensing and Transmitting Technology, Chongqing 400065, China; linjz@cqupt.edu.cn (J.L.); pangyu@cqupt.edu.cn (Y.P.); 3 International College, Chongqing University of Posts and Telecommunications, Chongqing 400065, China; 2015214742@stu.cqupt.edu.cn

**Keywords:** indoor positioning, Bluetooth, RSSI distance model, Kalman filter

## Abstract

The Global Navigation Satellite System (GNSS) cannot achieve accurate positioning and navigation in the indoor environment. Therefore, efficient indoor positioning technology has become a very active research topic. Bluetooth beacon positioning is one of the most widely used technologies. Because of the time-varying characteristics of the Bluetooth received signal strength indication (RSSI), traditional positioning algorithms have large ranging errors because they use fixed path loss models. In this paper, we propose an RSSI real-time correction method based on Bluetooth gateway which is used to detect the RSSI fluctuations of surrounding Bluetooth nodes and upload them to the cloud server. The terminal to be located collects the RSSIs of surrounding Bluetooth nodes, and then adjusts them by the RSSI fluctuation information stored on the server in real-time. The adjusted RSSIs can be used for calculation and achieve smaller positioning error. Moreover, it is difficult to accurately fit the RSSI distance model with the logarithmic distance loss model due to the complex electromagnetic environment in the room. Therefore, the back propagation neural network optimized by particle swarm optimization (PSO-BPNN) is used to train the RSSI distance model to reduce the positioning error. The experiment shows that the proposed method has better positioning accuracy than the traditional method.

## 1. Introduction

With the rapid development of mobile Internet technology, the demand for location-based services (LBS) has also increased. However, in indoor environments, satellite signals are easily occluded and there are serious multipath effects. Therefore, the positioning accuracy of GNSS is drastically reduced so that it can not meet the positioning requirements. With the emergence of a large number of indoor applications, such as the rapid search for parking spaces in underground parking lots and the search for elevators in large shopping malls, the acquisition of indoor location information is particularly important. People have gradually turned their attention to indoor positioning technology, hoping to solve this problem and achieve new breakthroughs [1]. A variety of indoor positioning technologies have been proposed, such as Bluetooth [2], ultra-wideband (UWB) [3], radio frequency identification (RFID) [4], micro-electro-mechanical system (MEMS) [5], wireless local area networks (WLAN) [6], computer vision [7], magnetic field [8], ultrasonic [9], infrared signal [10] and others.

Low-power Bluetooth is favored in indoor positioning because of its advantages such as easy deployment, low power consumption, and low cost. In 2013, Apple introduced the iBeacon technology based on Bluetooth low-energy (BLE), which made BLE widely used in various indoor environments. The positioning algorithms are mainly divided into two categories: the received signal strength indication (RSSI) distance method and wireless fingerprint positioning technology. Generally, the RSSI distance method acquires the received signal strength (RSS) from the Bluetooth anchor point to determine the distance-loss model, and then estimates the user’s position through some algorithms. Neburka [11] investigated the performance of BLE indoor positioning based on RSSI in ideal and real indoor environments. Wang [12] proposed a Bluetooth-based trilateration method employing the Bluetooth transmission model and trilateration positioning, which can only estimate an approximate region of the user. Kotanen [13] adopted an extended Kalman filter to process the Bluetooth signals and estimate the RSSI distance model. This method can obtain an accurate distance with the premise of precise received signal strength. However, in practical tests, the accuracy was still average. Shi [14] proposed a method of estimating the RSSI distance model using the back propagation neural network (BPNN), which improves the estimation accuracy of the distance-loss model but requires a large amount of data to train the neural network model. Zhou [15] employed the Kalman filter to process the collected RSSIs at first. The RSSI-distance model was obtained by curve fitting and the position was determined using the weighted least-squares algorithm. However, the RSSI fluctuation noise is not Gaussian in distribution which implies that the Kalman filter is obviously not optimal. Fingerprint localization is also a RSSI-based method [16,17]. It first collects all of the RSSIs of receivable Bluetooth beacons at the densest locations possible and then uses neural networks to match the locations with the RSSI. Finally, the user’s location can be estimated by the trained neural network. Pelant [18] evaluated BLE localization performances based on RSS fingerprinting mapped by propagation modes in an indoor environment. Liu [19] proposed a novel approach to track a user in an indoor environment by integrating a similarity-based sequence and dead reckoning. Zhou [20] proposed the use of a novel, improved, semi-supervised manifold alignment approach to reduce both the number of reference points (RPs) and the sampling time involved in the fingerprint construction. Fingerprint crowdsourcing has recently been promoted to relieve the burden of site surveying [21,22,23]. Wang [24] combined the fingerprinting-based and distance-based localization algorithms to improve performance. Rozum [25] proposed a new method to suppress RSSI fluctuation by employing spatial diversity and frequency diversity. Botta [26] proposed and analyzed the static calibration and dynamic calibration of a log-normal shadowing radio propagation model for the estimationof the distance between two wireless nodes. Li [27] proposed a method to adjust distance-loss model parameters dynamically. It utilizes the anchor nodes in the area to estimate the distance-loss model parameters in real-time cooperatively. The final location is calculated according to the trilateration or other positioning algorithms. However, the binary equation needs to be solved in real-time which has high complexity and brings about new problems caused by multiple approximations while eliminating a small amount of environmental impact.

The above methods may improve the accuracy of the distance estimation to a certain extent with the premire of fixed transmission power of Bluetooth beacons at one location. However, the transmission power fluctuates from time to time which results in an inaccurate distance-loss model and further inaccurate positioning. In order to solve this problem, this paper analyzes the time-varying characteristics of Bluetooth signal strength and proposes a method with real-time corrected RSSI to weaken the fluctuation influence. The contributions of this paper as follows:

- A RSSI Real-time Correction algorithm is proposed. A Bluetooth gateway is set at a fixed location and collects the RSSI of surrounding Bluetooth beacons to the server in real-time. Since the distance to the beacons is fixed, the server is able to estimate the fluctuation and feeds back to the user (mobile terminal) to correct the RSSI in real-time. Furthermore, the Kalman filtering is used to further smooth the RSSI.
- A back propagation neural network optimized by particle swarm optimization (PSO-BPNN) RSSI distance model is built. Then, the distance between the blind node and the anchor node is estimated using the RSSI distance model trained by PSO-BPNN.
- We perform an extensive experiment and the results show that the positioning error caused by the power fluctuation of the Bluetooth system is reduced obviously. The method does not need to spend a lot of time building a fingerprint database, and hence, it has low complexity. The experimental results show that the method has good localization accuracy and stability.

The rest of this paper is organized as follows. Section 2 introduces the related works. Section 3 describes the proposed system including the RSSI real-time correction algorithm as well as the PSO-BPNN RSSI distance model. In Section 4, the extensive experiments carried out in an actual indoor environment are described. Finally, Section 5 concludes the paper and provides some future directions.

## 2. Related Work

### 2.1. RSSI Error Distribution

**Figure 1:**
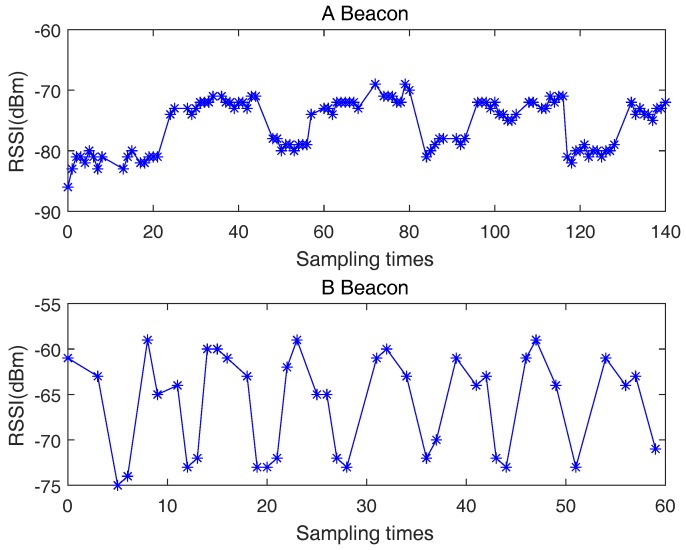
Bluetooth signal strength.

**Figure 2:**
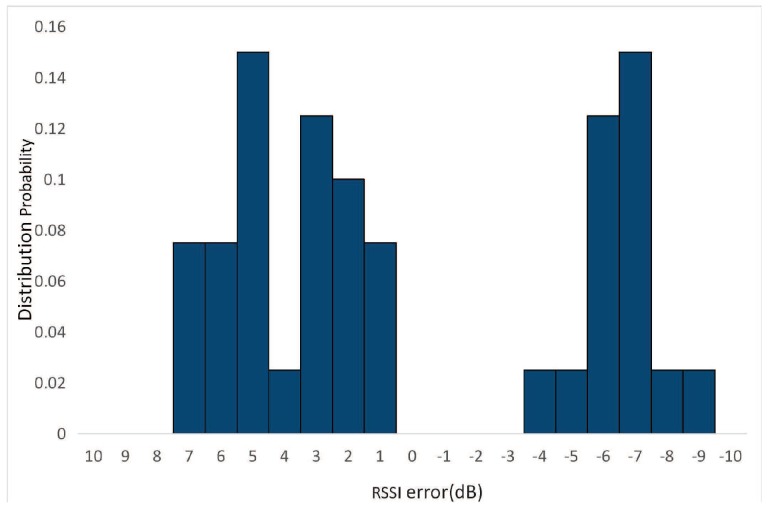
RSSI error distribution.

The RSSI measured by intelligent terminal contains complex noise, which seriously affects the positioning accuracy. It is difficult for Bluetooth system to transmit signals with fixed power, which results in time-varying characteristics of RSSI. Moreover, the indoor electromagnetic environment is complex, including multipath fading and other noise. We tested the RSSI fluctuation of two Bluetooth beacons produced by different companies, as shown in Figure 1. It can be seen that due to the instability of the Bluetooth system, both of the RSSI values are time-varying and the maximum fluctuation value is about 10 dB. In order to reduce the random fluctuation of the RSSI and improve the positioning accuracy, a sliding filter or an average filter is usually applied. However, in order to reduce power consumption, the frequency setting of Bluetooth is often low. Relying on multiple RSSI values to smooth means an increase in latency. This adds great difficulty to the implementation of a RSSI-based low-latency, high-precision positioning system [28]. The researchers tried to use a Kalman filter or improved Kalman filter to reduce the noise [29,30,31]. As presented in Figure 2, the measurement error in RSSI does not usually have a Gaussian distribution, so even with the adoption of a Kalman filter, it is difficult to achieve good results.

### 2.2. RSSI Distance Model

Common propagation path-loss models include the free space propagation model, the logarithmic distance path-loss model, etc. Studies have shown that the channel fading characteristic follows a lognormal distribution. RSSI distance measurement generally uses the logarithmic distance path-loss model [32,33,34]. It is expressed as

(1) $$RSSI=-10n\mathrm{lg}\left(\frac{d}{d_{0}}\right)+A+X_{\sigma },$$

where *d* is the distance between the transmitter and the receiver, and *n* is a path-loss parameter related to the specific wireless transmission environment. The more obstacles there are, the larger *n* will be. *A* is the RSSI with distance $d_{0}$ from the transmitter. $X_{\sigma }$ is a Gaussian-distribution random variable with mean 0 and variance $\sigma^{2}$.

For convenience of calculation, $d_{0}$ usually takes a value of 1 meter. Since $X_{\sigma }$ has a mean of 0, the distance-loss model can be obtained with

(2) $$\bar{RSSI}=-10n\log\left(d\right)+\bar{A},$$

where $\bar{A}$ is the average measured RSSI when the received node is 1 meter away from the transmit node which is related to the RF circuits of Bluetooth nodes. By gathering the RSSI values for Bluetooth beacons at different distances and using the least squares algorithm to fit the parameters, we can obtain the RSSI distance model.

It can be seen from (2) that parameters *A* and *n* need to be accurately estimated in order to improve the ranging accuracy. Parameter *n* is related to the wireless transmission environment and can be obtained by fitting a large number of experimental measurements. *A* is determined by the Bluetooth transmit power. Ideally, the value of *A* for one Bluetooth beacon should be fixed. In reality, the Bluetooth transmit power has time-varying characteristics. It is difficult to calculate the relationship between RSSI and the distance accurately by using the logarithmic distance loss model because of the complex indoor environment. Researchers have used the neural network to fit the RSSI distance model. However, since the BP neural network is prone to converging to a local minimum point [35,36], it is difficult to obtain a good indoor RSSI distance model. The use of the particle swarm optimization algorithm (PSO) to optimize the weights and thresholds of the BP neural network can effectively prevent the BP neural network from falling into the local optimal solution and reduce the convergence time [37,38].

## 3. RSSI Real-Time Correction Algorithm

**Figure 3:**
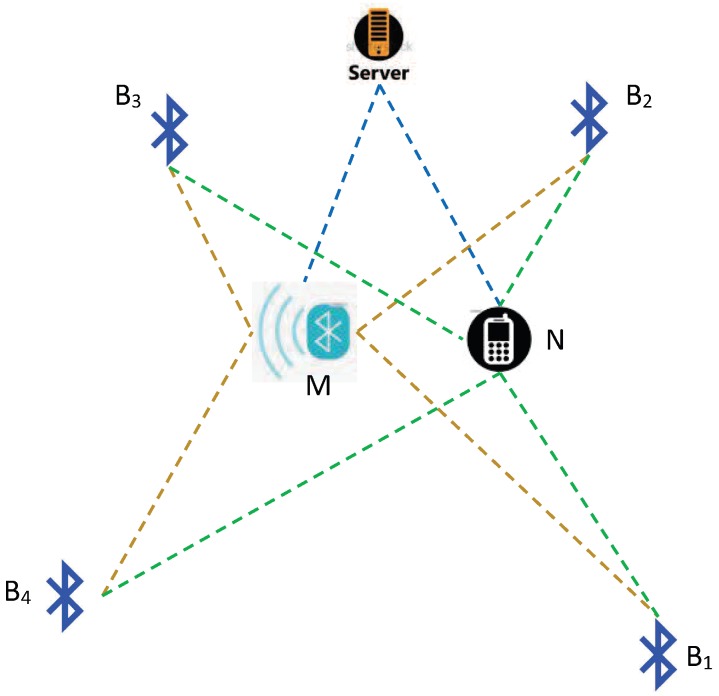
Diagram of indoor positioning by Bluetooth nodes with received signal strength indication (RSSI) correction.

In order to reduce the positioning error caused by RSSI fluctuation, we monitored the RSSI fluctuation in real-time through the Bluetooth gateway and compensated for the RSSI measured by the blind nodes. Our system model is shown in Figure 3, where $B_{1}$, $B_{2}$, $B_{3}$ and $B_{4}$ are the Bluetooth anchor nodes, *M* is a Bluetooth gateway, and *N* is a blind node which is usually a user or mobile terminal.

In the offline phase, the average RSSI is recorded at different distances, and the corresponding average RSSI distance model can be obtained by curve fitting as

(3) $$\bar{RSSI}=-10n\mathrm{lg}\left(d\right)+\bar{A_{l}},$$

where $A_{l}$ is the average RSSI at a distance of 1 m from $B_{l}$. Since *M*, *N*, and *B* are in a relatively small range, we can assume that the transmission environment among them is approximately the same, which means that the loss parameter (*n*) is the same. In this way, *n* and $\bar{A_{l}}$ are determined.

In the online phase, the server records $R_{Ml}$ and takes the average time taken to achieve the average signal strength ($\bar{R_{Ml}}$) at regular intervals. Based on ref. (3), we obtain

(4) $$\bar{R_{Ml}}=-10n\mathrm{lg}\left(d_{l}\right)+\bar{A_{l}}.$$

Assume the distance between gateway *M* and Bluetooth node $B_{l}\;\left(l=1,2,3,4\right)$ is $d_{l}$. First, *M* collects the RSSI $R_{Ml}$ of the surrounding Bluetooth node ($B_{l}$) and uploads them to the server through an wired or wireless Internet interface in real-time. The real-time RSSI distance model is

(5) $$R_{Ml}=-10n\mathrm{lg}\left(d_{l}\right)+A_{l},$$

where $A_{l}$ is the RSSI at a distance of 1 m from $B_{l}$.

Similarly, for the blind node (*N*) to be located, the average signal strength ($\bar{R_{Nl}}$) of node $B_{l}$ and the real-time signal strength ($R_{Nl}$) are represented as follows:

(6) $$\bar{R_{Nl}}=-10n\mathrm{lg}\left(d_{Nl}\right)+\bar{A_{l}}$$

(7) $$R_{Nl}=-10n\mathrm{lg}\left(d_{Nl}\right)+A_{l}.$$

Using (4) and (5), we obtain

(8) $$R_{Ml}-\bar{R_{Ml}}=A_{l}-\bar{A_{l}}=\Delta A_{l},$$

where $\Delta A_{l}$ represents the real-time fluctuation of the Bluetooth system which we call the RSSI correction offset.

Combining (6) and (7) gives

(9) $$\begin{array}{c} R_{Nl}-\bar{R_{Nl}}=A_{l}-\bar{A_{l}}\\ R_{Nl}=\Delta A_{l}+\bar{R_{Nl}}. \end{array}$$

Further, the corrected RSSI ${\tilde{R}}_{Nl}$ is obtained as

(10) $$\begin{array}{cc} {\tilde{R}}_{Nl} & =R_{Nl}-\Delta A_{l}\\ & =-10n\log\left(d_{Nl}\right)+\bar{A_{l}}, \end{array}$$

which is also the real-time path-loss model.

Through the above algorithm, we can eliminate the error caused by the fluctuation of Bluetooth transmit power and get a more accurate RSSI distance model. It is crucial for precise indoor positioning.

## 4. System Model

### 4.1. Positioning Step

**Figure 4:**
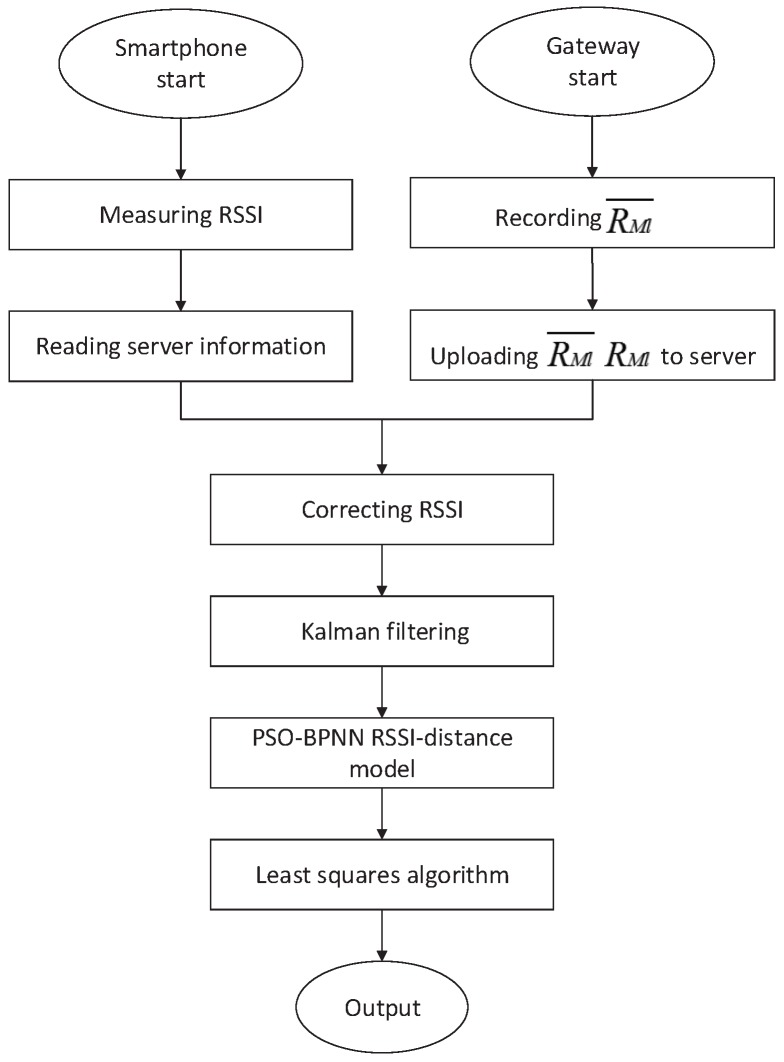
The positioning schematic diagram.

According to the above analysis, the position of the Bluetooth gateway is arbitrary, and it is only required for the anchor node to communicate with at least one Bluetooth gateway. Based on the architecture of the positioning system in Figure 4, we propose the following positioning method:

1. Deploy anchor nodes and Bluetooth gateways;
2. The Bluetooth gateway measures the real-time signal strength of each anchor node and uploads it to the server;
3. The server records the mean signal strength of each anchor node and calculates $\Delta A_{n}$;
4. The blind node measures the signal strength of each anchor node, reads the server information, and corrects the RSSI according to (9);
5. The corrected RSSI is smoothed by the Kalman filter and RSSI to is converted to distance using the PSO-BPNN model (15);
6. The blind node position is estimated using the least squares algorithm.

### 4.2. PSO-BPNN RSSI Distance Model

**Figure 5:**
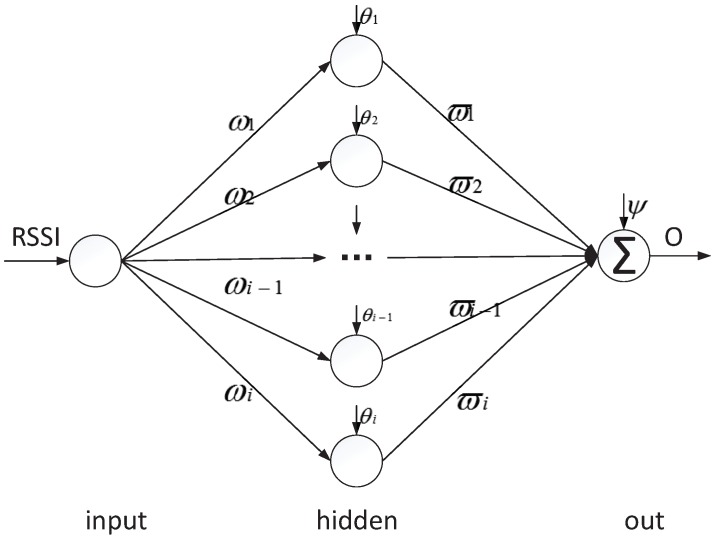
Neural network structure.

It is difficult to accurately map the relationship between RSSI and distance with the logarithmic distance loss model. In this section, the PSO-BPNN Algorithm 1 is used to train the RSSI distance model. The PSO-BPNN is composed of a three-layer structure, including one input node, *i* hidden nodes, and one output node. The training sample input value is $RSSI=\left\{RSSI_{1},\cdots ,RSSI_{j},\cdots ,RSSI_{n}\right\}$. The training sample output value is $D=\left\{d_{1},\cdots ,d_{j},\cdots ,d_{n}\right\}$. As shown in Figure 5, $\omega_{i}$ is defined as the weights from the input layer to the hidden layer, $\theta_{i}$ is defined as the threshold value of the hidden layer node, $\varpi_{j}$ is defined as the weights from the hidden layer to the output layer, ψ is defined as the threshold value of the output layer node, and *O* is defined as the output of the neural network.

The PSO preliminarily optimizes the parameters of BPNN to avoid the BPNN falling into the local optimal solution. In the PSO algorithm, the potential solution of each optimization problem is imagined as a point in a x-dimensional space which is called a particle. The particle moves around in the search space according to simple mathematical formulae regarding the particle’s position and velocity. Each particle’s movement is also influenced by its local best known position and the global best known position, which are found by the fitness function. After many iterations, the optimal positions of the particles and the extremum of the fitness function are calculated. In this paper, the fitness function is shown as

(11) $$\begin{array}{c} Fit=\sum_{i=1}^{n}d_{i}-o_{i}. \end{array}$$

The PSO iteration formula is

(12) $$\begin{array}{c} v_{i,d}^{k+1}=uv_{i,d}^{k}+c_{1}r_{1}\left(p_{i,d}-x_{i,d}^{k}\right)+c_{2}r_{2}\left(p_{g,d}-x_{i,d}^{k}\right)\\ x_{i,d}^{k+1}=x_{i,d}^{k}+v_{i,d}^{k+1}, \end{array}$$

where $x_{i,d}=\left\{\omega_{1},\cdots \omega_{i},\theta_{i},\cdots \theta_{i},\cdots \varpi_{1},\cdots \varpi_{i},\cdots \psi \right\}$ are the particles. *u* means the inertia weight, $r_{1}$ and $r_{2}$ are two random numbers in the range [0, 1]. $c_{1}$ and $c_{2}$ are two positive constants which is called learning factor or accelerating factor. $c_{1}$ is used to adjusted the step length of the particle flying to its own best position. $c_{2}$ is used to adjusted the step length of the particle flying to the swarm’s best position. $p_{i,d}$ is the optimal value searched by the *i*-th particle. $p_{g,d}$ is the optimal value searched by the whole particle swarm.

Neural network weights and thresholds can be optimized through multiple iterations. The optimized weights and thresholds are assigned to the BPNN to further train the network model. The hidden layer output is (13)

(13) $$\begin{array}{c} H_{i}=tansig\left(\sum_{i=1}^{n}\omega_{i}x_{i}+\theta_{i}\right). \end{array}$$

The output layer output is (14)

(14) $$\begin{array}{c} O=purelin\left(\sum_{i=1}^{l}Hi\varpi i+\psi \right). \end{array}$$

**Algorithm 1** PSO-BPNN algorithm
1:Initialize the BPNN parameters: training samples, hidden layer, learning rate2:**for** each particle i=1,⋯,ς **do**3: Initialize the PSO parameters: $c_{1},c_{2},x_{i},$4: Initialize the particle’s best known position to its initial position: $p_{i}$←$x_{i}$5: **if** $p_{i}<p_{g}$ **then**6:  Update the swarm’s best known position: $p_{g}\leftarrow p_{i}$7: **end if**8: Initialize the particle’s velocity: $v_{i}$9:
**end for**
10:**while** a termination criterion is not met do **do**11: **for** each particle i=1,⋯,ς **do**12:  **for** each dimension d=1,…,ι **do**13:   Update the particle’s velocity: $v_{i,d}$14:  **end for**15:  Update the particle’s position: $x_{i}$16: **end for**17: **if** $Fit(x_{i})$ < $Fit(p_{i})$ **then**18:  Update the particle’s best known position: $p_{i}\leftarrow x_{i}$19: **end if**20: **if** $Fit(p_{i})$ < $Fit(p_{g})$ **then**21:  Update the particle’s best known position: $p_{g}\leftarrow p_{i}$22: **end if**23:
**end while**
24:Update the parameters of BPNN based on $p_{g}$25:Train the BPNN based on (13) and (14)26:**return** PSO-BPNN model


Using PSO-BPNN, we can get a more accurate RSSI-distance model, which can be expressed as

(15) $$\begin{array}{c} RSSI\to d \end{array}$$

### 4.3. Kalman Filter

Besides the power fluctuation, there are some other noises that exist after RSSI correction with (9). The Kalman filter is an effective algorithm for solving the problem. In this subsection, the state equation is expressed as

(16) $$\begin{array}{c} x_{k}=x_{k-1}+w_{k-1}, \end{array}$$

where $w_{k-1}$ is the state model noise with variance *Q*. Accordingly, the observation equation can be given as

(17) $$\begin{array}{c} z_{k}=x_{k}+v_{k}, \end{array}$$

where $z_{k}\in R^{2}$ contains the corrected RSSI, and $v_{k}$ is the observation noise with variance *R*. The renewal equation can be further obtained as

(18) $$\begin{array}{c} {{\hat{x}}_{k}}^{-}={\hat{x}}_{k-1}\\ {P_{k}}^{-}=P_{k-1}+Q\\ K_{k}={P_{k}}^{-}/\left({P_{k}}^{-}+R\right)\\ {\hat{x}}_{k}={{\hat{x}}_{k}}^{-}+K_{k}\left(z_{k}-{{\hat{x}}_{k}}^{-}\right)\\ P_{k}=\left(1-K_{k}\right){P_{k}}^{-}, \end{array}$$

where ${{\hat{x}}_{k}}^{-}$ is the predicted state estimation, ${P_{k}}^{-}$ denotes the predicted error covariance, $K_{k}$ represents the optimal Kalman gain, ${\hat{x}}_{k}$ is the optimal state estimation, and $P_{k}$ means the updated estimation covariance. After smoothing the corrected RSSI values with the Kalman filter, we can achieve more accurate distance estimation between the blind node and the anchor node based on (15).

### 4.4. Least Squares Algorithm

The least squares algorithm is a standard approach in regression analysis that is used to approximate the solutions of overdetermined systems. Compared with the triangular centroid algorithm, the least squares algorithm can estimate the locations of blind nodes more accurately. Suppose there are m(m≥3) anchor nodes with coordinates $\left(x_{1},y_{1}\right),\left(x_{2},y_{2}\right),\ldots ,\left(x_{m},y_{m}\right)$, and blind nodes with coordinates (x,y). We can obtain

(19) $$\begin{array}{c} \left\{\begin{array}{c} {\left(x_{1}-x\right)}^{2}+{\left(y_{1}-y\right)}^{2}={d_{1}}^{2}\\ {\left(x_{2}-x\right)}^{2}+{\left(y_{2}-y\right)}^{2}={d_{2}}^{2}\\ \vdots \\ {\left(x_{m}-x\right)}^{2}+{\left(y_{n}-y\right)}^{2}={d_{m}}^{2}. \end{array}\right. \end{array}$$

By subtracting the *m*-th equation from the first m−1 equation in (19), the linear Equation (20) is obtained:

(20) $$\begin{array}{c} AX=b \end{array}$$

(21) $$\begin{array}{c} A=\left(\begin{array}{cc} 2(x_{1}-x_{m}) & 2(y_{1}-y_{m})\\ 2(x_{2}-x_{m}) & 2(y_{2}-y_{m})\\ \vdots & \vdots \\ 2(x_{m-1}-x_{m}) & 2(y_{m-1}-y_{m}) \end{array}\right)\; \end{array}$$

(22) $$\begin{array}{c} b=\left(\begin{array}{c} {x_{1}}^{2}-{x_{m}}^{2}+{y_{1}}^{2}-{y_{m}}^{2}+{d_{1}}^{2}-{d_{m}}^{2}\\ {x_{2}}^{2}-{x_{m}}^{2}+{y_{2}}^{2}-{y_{m}}^{2}+{d_{2}}^{2}-{d_{m}}^{2}\\ \vdots \\ {x_{m-1}}^{2}-{x_{m}}^{2}+{y_{m-1}}^{2}-{y_{m}}^{2}+{d_{m-1}}^{2}-{d_{m}}^{2} \end{array}\right).\; \end{array}$$

Finally, we can get the blind node’s position (*X*):

(23) $$\begin{array}{c} X={\left(A^{T}A\right)}^{-1}A^{T}b. \end{array}$$

## 5. Experiment

**Figure 6:**
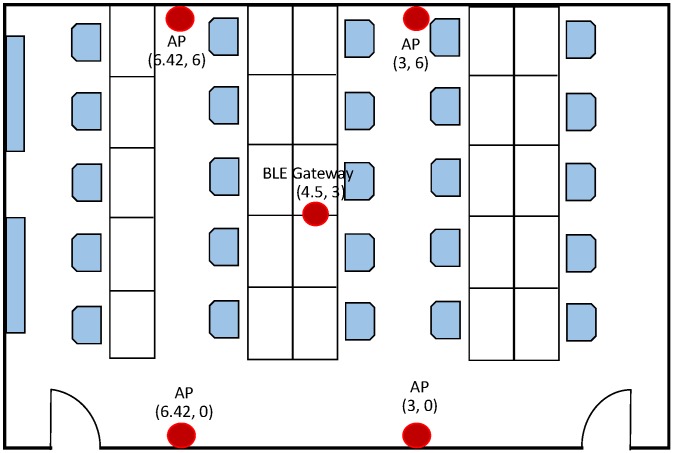
Experimental environment.

In this paper, the wireless network was composed of four BLE beacons (Bright Beacon), an Android terminal, and one gateway (Bright Beacon). As shown in Figure 6, we carried out the localization experiment in an office of 9 m×6 m with multiple people working, including cubicles, bookcases, computers, etc.

**Figure 7:**
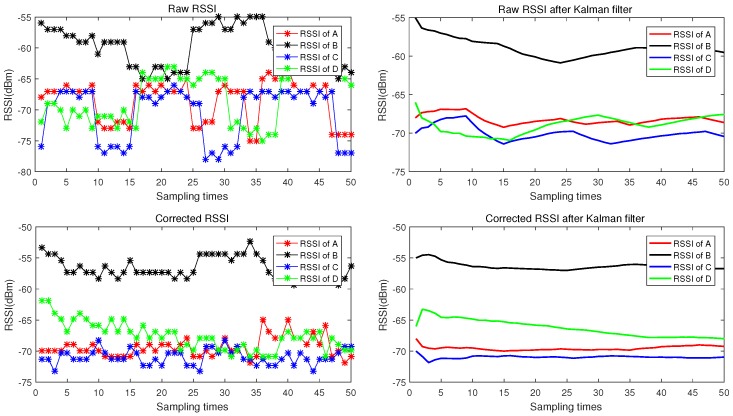
Comparison of the collected RSSI values before and after Kalman filtering.

We placed four BLE beacons at the same height with coordinates A(3,6), B(3,0), C(6.42,6), and D(6.42,0), and then placed the Bluetooth gateway at a fixed location. The blind node coordinates were (2.67,2). The transmission interval of the beacon frame of all of the four Bluetooth nodes was set to 500 ms, and 50 times of RSSI were collected continuously. The raw RSSI values collected by the Android terminal and the corrected RSSI by the Bluetooth gateway are shown in Figure 7.

**Figure 8:**
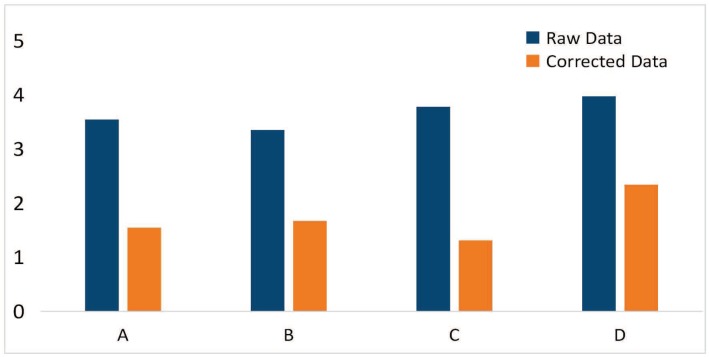
The standard deviation of the RSSI before and after correction.

It can be seen that the original RSSI data has a sharp fluctuation and presents a very obvious time-varying characteristic. Even after Kalman filtering, the data still has a great fluctuation. The RSSI data after corrected by the information from the Bluetooth gateway is more stable by eliminating part effects of the signal fluctuation. After further noise elimination using Kalman filter, we get smoother data. Figure 8 shows that the standard deviation(SD) of RSSI data after corrected by the Bluetooth gateway is greatly reduced, which means a more precise distance can be achieved between the anchor node and the blind node.

**Figure 9:**
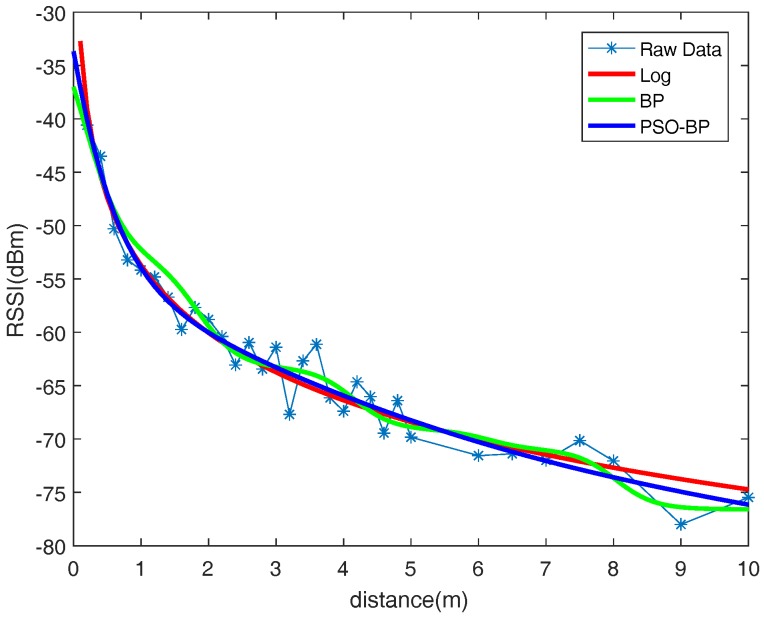
The result of RSSI distance model fitted by the logarithmic distance loss model, back propagation neural network (BPNN), and back propagation neural network optimized by particle swarm optimization (PSO-BPNN).

**Figure 10:**
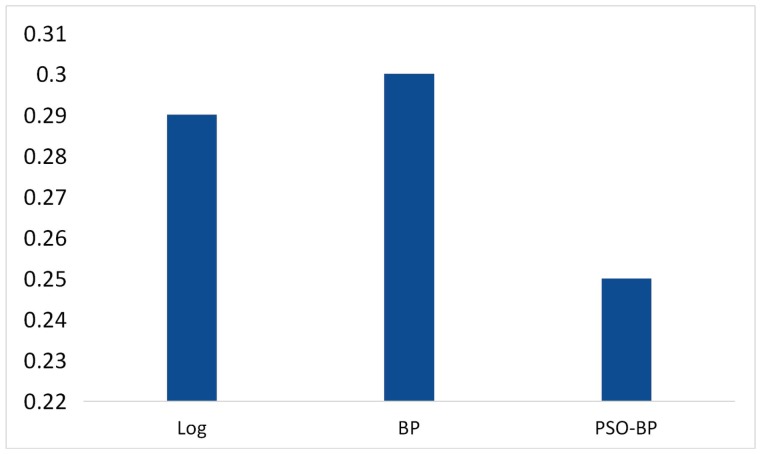
The normalized sum of squared errors (SSE) of the RSSI distance model fitted by the logarithmic distance loss model, BPNN, and PSO-BPNN.

In this paper, three methods were tested to fit the RSSI distance model, including the logarithmic distance loss model, BPNN, and PSO-BPNN. In the test, BPNN and PSO-BPNN adopted a single hidden layer network with five hidden layer nodes. The results of the tests are given in Figure 9. Figure 10 shows the normalized sum of squared errors (SSE) of the RSSI distance model fitted by three methods . The SSE of the logarithmic distance model fitting with the least squares algorithm was 0.29. The SSE of the RSSI distance model trained by the BP neural network was 0.30. The RSSI distance model trained by PSO-BPNN had the lowest error rate—the error rate was only 0.25—and it showed good robustness.

**Figure 11:**
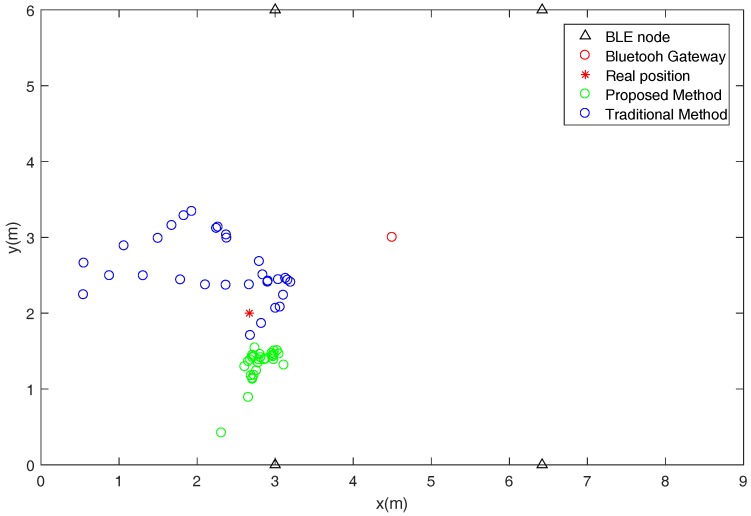
Comparison of the positioning results between the traditional and proposed methods.

The results of the indoor positioning experiment are shown in Figure 11. Since the noise caused by the RSSI fluctuation cannot be eliminated, the traditional Kalman filtering method has great positioning error. By employing the Bluetooth gateway to correct RSSI in real-time, and further using PSO-BPNN to train the RSSI distance model, better positioning results were achieved. In 30 consecutive positioning tests, our method showed more stable positioning.

**Figure 12:**
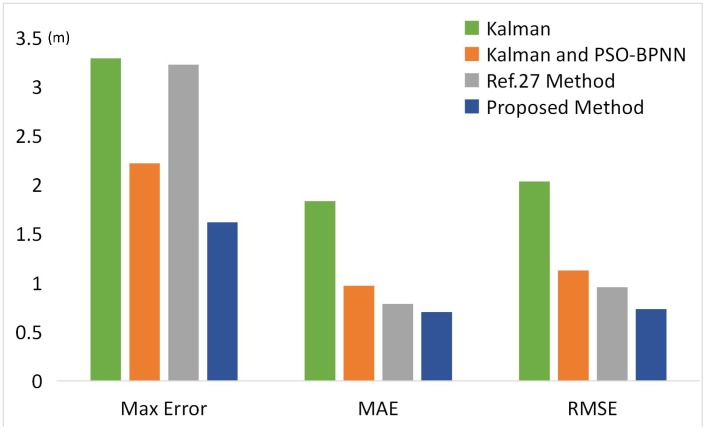
Comparison of the positioning RMSE, MAE ,and maximum error between the traditional and proposed methods.

We randomly selected eight test points in the office, and their positioning errors are shown in Figure 12. We can see that due to the fluctuation of the Bluetooth system, the positioning results were still poor even when the Kalman Filter was used. The root mean square error (RMSE) was 2.0361 m, the mean absolute error (MAE) was 1.8357 m, and the maximum positioning error was 3.29 m. The Kalman filter and PSO-BPNN RSSI distance model were able to effectively reduce the positioning error— the RMSE was 1.1279 m, the MAE was 0.9704 m, and the maximum positioning error was 2.21 m.

The method proposed in ref. [27] was able to reduce the error caused by the RSSI fluctuation, but it brings new error due to the approximate nature of the calculation. The RMSE was 0.9583 m, the MAE was 0.7882 m, and the maximum positioning error was 3.23 m. After correcting the RSSI and accurately estimating the RSSI distance model by the proposed method, the positioning accuracy improved obviously. The RMSE was only 0.7343 m, the MAE was 0.7018 m, and the maximum positioning error was only 1.61 m. The results show that the noise caused by the RSSI fluctuation was reduced and our algorithm provided a good positioning result.

## 6. Conclusions and Future Work

This paper presents an indoor location method for the dynamic correction of RSSI by deploying the Bluetooth gateway. The location of the Bluetooth gateway is not strictly required; it is only required that each anchor node can communicate with at least one Bluetooth gateway. In addition, we used the PSO-BPNN trained RSSI-distance model in the offline stage. Then, we used the proposed algorithm to correct the RSSI in real-time and used the Kalman filter to further smooth the data. After smoothing the RSSI, the distance between the blind node and the BLE node was estimated by the PSO-BPNN RSSI distance model. Finally we used the least squares algorithm to estimate the terminal position. The experimental results show that the algorithm can improve the positioning accuracy and meet the requirements of the indoor positioning system. In this paper, some special positions were not considered, like indoor corners and other complex electromagnetic regions. Moreover, it is difficult to obtain continuous positioning with high precision only by BLE positioning technology. Therefore, the proposed method can be further improved by fusion positioning with multiple sensors which could be investigated in the future, including the positioning in some special cases.
